# Pre-Existing T- and B-Cell Defects in One Progressive Multifocal Leukoencephalopathy Patient

**DOI:** 10.1371/journal.pone.0034493

**Published:** 2012-04-04

**Authors:** Alessandra Sottini, Ruggero Capra, Cinzia Zanotti, Marco Chiarini, Federico Serana, Doris Ricotta, Luigi Caimi, Luisa Imberti

**Affiliations:** 1 Biotechnology Laboratory, Diagnostics Department, Spedali Civili of Brescia, Brescia, Italy; 2 Multiple Sclerosis Center, Spedali Civili of Brescia, Montichiari, Brescia, Italy; 3 Department of Biomedical Sciences and Biotechnologies, University of Brescia, Brescia, Italy; National Institutes of Health, United States of America

## Abstract

Progressive multifocal leukoencephalopathy (PML) usually occurs in patients with severe immunosuppression, hematological malignancies, chronic inflammatory conditions or receiving organ transplant. Recently, PML has also been observed in patients treated with monoclonal antibodies. By taking advantage of the availability of samples from a multiple sclerosis (MS) patient treated with natalizumab, the antibody anti-α4 integrin, who developed PML and was monitored starting before therapy initiation, we investigated the fate of T and B lymphocytes in the onset of PML. Real-time PCR was used to measure new T- and B-cell production by means of T-cell receptor excision circle (TREC) and K-deleting recombination excision circle (KREC) analysis and to quantify transcripts for CD34, terminal-deoxynucleotidyltransferase, and V pre-B lymphocyte gene 1. T- and B-cell subsets and T-cell heterogeneity were measured by flow cytometry and spectratyping. The data were compared to those of untreated and natalizumab-treated MS patients and healthy donors. Before therapy, a patient who developed PML had a low TREC and KREC number; TRECs remained low, while KRECs and pre-B lymphocyte gene 1 transcripts peaked at 6 months of therapy and then decreased at PML diagnosis. Flow cytometry confirmed the deficient number of newly produced T lymphocytes, counterbalanced by an increase in TEMRA cells. The percentage of naive B cells increased by approximately 70% after 6 months of therapy, but B lymphocyte number remained low for the entire treatment period. T-cell heterogeneity and immunoglobulins were reduced.

Although performed in a single patient, all results showed that an immune deficit, together with an increase in newly produced B cells a few months after therapy initiation, may predispose the patient to PML. These findings indicate the TREC/KREC assay is a potential tool to identify patients at risk of developing PML and may provide insights into the immunological involvement of monoclonal antibody-associated therapies.

## Introduction

Progressive multifocal leukoencephalopathy (PML) is a rare, but often fatal, demyelinating brain disease caused by the JC virus (JCV) [1] that usually occurs in patients with severe immunosuppression. Prior to the era of the HIV epidemic, PML arose preferentially in a few immunosuppressed patients, including organ transplant recipients and those with hematological malignancies and chronic inflammatory conditions. Recently, PML has also been observed in patients treated with monoclonal antibodies (MoAb) [2], [3]. MoAb-associated PML is likely the result of a complex combination of several pathogenic mechanisms, including mobilization of JCV-carrying CD34^+^ hematopoietic stem cells and pre-B cells, neurotropic JCV transformation, changes in the blood-brain barrier/central nervous system (CNS) parenchyma [4]–[9], and alterations in peripheral cell-mediated immunity, with failure of CD4 and CD8 cells and specific anti-JCV antibodies to control JCV-induced PML [10]–[13].

The estimated overall risk of PML in natalizumab-treated multiple sclerosis (MS) patients is 1.51 per 1000 patients and this risk might increase beyond 24 months of treatment [14], [15]. The mechanisms leading to natalizumab-induced PML are still incompletely understood; however, because the blocking of the α4 chain of VLA-4 (α4β1) and α4β7 integrins [16] prevents adhesion and diapedesis of activated lymphocytes through the blood-brain barrier [17], this drug might also block the entry of JCV-specific T cells, resulting in decreased nervous system immune surveillance [18]. Furthermore, circulating B cells, and especially pre-B cells, are elevated in natalizumab-treated patients, raising the possibility that the therapeutic effects, as well as the side effects, of natalizumab can be mediated, at least in part, by the action of B cells [19]. However, a recent study demonstrated that CD34^+^ cells are not a relevant reservoir for JCV DNA in patients treated with natalizumab [20] and, therefore, alternative sites or mechanisms of brain infection should be considered. Concerning the immune response against JCV, the results are conflicting because there are studies that do not find alterations in the immune response to JCV, whereas others demonstrate a reduced T-cell activity during natalizumab treatment [21], [22]. Therefore, the understanding of the biological basis of natalizumab-induced PML development is presently undefined, mostly due to the lack of long-term follow-up studies aimed at analyzing laboratory parameters that may help to recapitulate the onset of the disease and, possibly, to prevent it. We performed the present study with the goal of monitoring the humoral and cellular response before and after natalizumab therapy in a MS patient who developed PML at the 34^th^ month of natalizumab therapy (pmlMS). MS patients treated with natalizumab but without PML (nMS), untreated patients (uMS), and healthy donors (HD) were used as controls.

## Materials and Methods

### Ethics Statement

All the archived samples employed in this study have been handled according to the institutional guidelines, as recommended by the declaration of Helsinki. Written informed consent was obtained from all participants. The study has been reviewed and approved by the Medical Ethics Committee of the Azienda Ospedaliera Spedali Civili of Brescia.

### Patients and sample collection

We investigated the immune status of six patients with relapsing-remitting MS, who were treated with natalizumab because of frequent relapses during treatments with other immunomodulatory agents (listed in Table 1). After 34 months of therapy, a 42-year-old woman developed PML, which was diagnosed on the basis of clinical presentation, magnetic resonance images, and the presence of 24 copies/mL of JCV (measured at LMMN/NINDS/NIH) in cerebrospinal fluid (CSF). She did not show clinical signs of immune reconstitution inflammatory syndrome after drug suspension and plasmapheresis.

**Table 1:** Natalizumab-treated patient characteristics.

		age				available samples - time points					
		at disease onset	at the first sample	disease duration	therapies before	T0	T1	T2	T3	T4	EDSS**
	gender	(y)	(y)	(y)	natalizumab*	(months of natalizumab therapy )					at T0
pmlMS	F	16	39	26	IFNβ 1a (30 µg/w; 22 and 44 µg 3 times/w); MTX (120 mg/m^2^)	0	6	12	15	34***	7
nMS1	F	12	17.2	7	IFNβ 1a (30 µg/w; 22 and 44 µg 3 times/w?)	0	6	12	15	35	2.5
nMS2	F	31	32	5	IFNβ 1a (44 µg 3 times/w)	0	6	12	21	36	4
nMS3	F	29	41	15	IFNβ 1a (44 µg/w); IFNβ 1b; MTX (90 mg/m^2^); GA (20 mg/day)	0	6	12	na	32	4
nMS4	M	25	37	12	IFNβ 1a (30 µg/w and 22 µg 3 times/w); MTX (110 mg/m^2^)	0	6	12	15	40	6
nMS5	M	23	37	17	IFNβ1a (22 µg 3 times/w)	0	6	12	15	27	4.5

Only the long-lasting and relevant therapies are reported. The interval between IFNβ 1a or GA therapies and natalizumab was at least of 30 days. plmMS received MTX from February 2004 to November 2006, nMS3 from February 2003 to March 2005 and nMS4 from July 2004 to February 2007.

** There was no trend of EDSS up- or downwards.

*** Time point in which PML was diagnosed. Four different samples were obtained over a 1-month period. For the other time points and in all other patients, only one biological sample was analyzed.

F, female; M, male; y, years; w, weeks; IFN, interferon; MTX, mitoxantrone; GA, glatiramer acetate; na, not available.

Peripheral blood, drawn into PAXgene tubes (PreAnalytiX, Hombrechtikon, Germany) for RNA preparation, into heparinized tubes for peripheral blood mononuclear cell (PBMC) isolation, and into tubes without anticoagulant for immunoglobulin (Ig) dosage by nephelometry, was obtained from the following: 6 MS patients before the first infusion of natalizumab (T0) and at the time points indicated in Table 1; 18 uMS patients (5 males, median age 41 years, range 33–47; and 13 females, median age 34 years, range 21–46); and from 24 age- and gender-matched HD (7 males, median age 38 years, range 24–42; and 17 females, median age 35 years, range 26–45). PBMCs were isolated by Ficoll-Hypaque density gradient centrifugation, washed twice in PBS, counted, separated in 3×10^6^ to 5×10^6^ cell aliquots, frozen in dimethyl sulfoxide solution, and stored in liquid nitrogen.

### Cytofluorimetric characterization of T- and B-lymphocyte subpopulations

T- and B-cell subsets were determined by six-color cytofluorimetric analysis. Briefly, phycoerythrin anti-CD3, allophycocyanin-H7 anti-CD4, phycoerythrin-Cy7 anti-CD8, fluorescein isothiocyanate anti-CD45RA (BD Pharmingen, Heidelberg, Germany), peridin-clorophyll protein-Cy5.5 anti-CCR7 (BioLegend, San Diego, CA), and allophycocyanin anti-CD31 (Miltenyi, Bergisch Gladbach, Germany) MoAb were used for T-cell subpopulation characterization. For B-cell analysis, PBMCs were stained with different amount of peridin-clorophyll protein-Cy5.5 anti-CD19, allophycocyanin anti-CD21, fluorescein isothiocyanate anti-IgD, and phycoerythrin anti-CD27 (BD Pharmingen) MoAb. The data were collected using a six-color 2-laser BD FACSCanto II cytometer and analyzed with FACS Diva software (BD Biosciences, San Jose, CA).

### Real-time PCR for T-cell receptor excision circles (TRECs) and K-deleting recombination excision circles (KRECs), CD34, terminal deoxynucleotidyltransferase (DNTT), and V pre-B lymphocyte gene 1 (Vpreβ1) transcripts

TREC and KREC molecules were measured by duplex real-time PCR in DNA extracted from PBMCs using the QIAamp DNA Blood Mini Kit (Qiagen, Valencia, CA). The sequence of primers and probes for TRECs, KRECs, and for the reference gene, which was a fragment of T-cell receptor (TCR) alpha constant (TCRAC) gene, together with the real-time protocol, were previously described [23]. The number of the target molecules in the samples was extrapolated by the standard curve obtained by serial dilutions (from 10^6^ to 10 copies) of a linearized plasmid DNA, containing inserts corresponding to fragments of TRECs, KRECs, and TCRAC, which were amplified in each PCR plate. TRECs and KRECs were expressed per mL of blood because their number was corrected for lymphocyte-monocyte count in 1 mL of blood.

For CD34, DNTT, and Vpreβ1 RNA quantification, RNA and cDNA were prepared using a PAXgene Blood RNA kit (PreAnalytiX) and Taqman reverse transcription reagents (Applied Biosystems, Foster City, CA), respectively. Primers and probes were bought from Applied Biosystems (CD34: Hs00156373_m1, DNTT: Hs00172743_m1, and Vpreβ1: Hs00356766_g1) or synthesized according to the Applied Biosystems recommendations (GAPDH forward primer: 5′GAAGGTGAAGGTCGGAGTC3′, reverse primer: 5′GAAGATGGTGATGGGATTTC3′, and probe: Fam-CAAGCTTCCCGTTCTCAGCC-Tamra). All of these genes were analyzed on the 7500 Fast real-time PCR system (Applied Biosystems) along with day-to-day controls.

### TCR beta variable (TCRBV) complementarity determining region-3 (CDR3) size spectratyping

Total RNA was prepared using the PAXgene Blood RNA kit (PreAnalytiX). The first strand of the TCR beta (TCRB) chain-specific complementary DNA, synthesized using a reverse primer specific for both TCRB constant (TCRBC) region genes 1 and 2 (5′ATCTCTGCTTCTGATGGCTCAAA3′), and TaqMan Reverse transcription reagents (Applied Biosystems), was immediately utilized for a TCRB variable (TCRBV) gene multiplex PCR that allows detection of the 23 functional TCRBV families, as described by Akatsuka et al [24]. Fluorescent PCR products were electrophoresed on an ABI 3130 analyzer (Applied Biosystems). Peak number, size, and height, as well as the area under the curve of the electropherogram, were calculated using Peak Scanner software version 1 (Applied Biosystems). The relative expression of each TCRBV transcript was quantified according to the formula described in Muraro et al [25] and the percentage area corresponding to each CDR3 peak in the TCRBV spectrum of lengths as well as the measure of perturbations were calculated according to Gorochov et al [26].

### Statistics

Because only one patient with PML was available, measures of her immune parameters were considered statistically different from those of nMS and uMS patients or HD samples (used as controls) when the values were outside the confidence interval of their respective means. To account for the multiple comparisons involving the same datasets, Bonferroni corrected P-values were employed. In particular, for TRECs and KRECs, the corrected P was 0.01, thus the 99% confidence intervals were calculated (after log transformation of data), while, for flow cytometry data, to account for 15 multiple comparisons, the corrected P-value was 0.0033, and the 99.6% confidence intervals were obtained. Comparisons between the nMS, uMS, and HD means were performed by two-tailed t-tests, with a Bonferroni corrected p-value of P = 0.01.

## Results

### Analysis of new T- and B- lymphocyte production by means of TREC and KREC quantification

**Figure 1:**
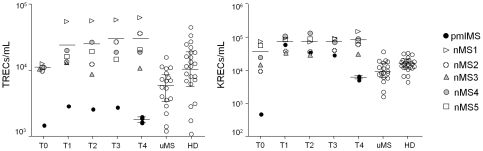
Levels of TRECs and KRECs. Levels of TRECs and KRECs in patient who developed PML (pmlMS; black dots), natalizumab-treated patients (nMS), untreated patients (uMS; white dots), and healthy donors (HD; white dots) were reported. The indicated time points (T0, T1, T2, T3, and T4) were those described in Table 1. In the pmlMS patient, whose four samples were analyzed at the time point T4, TRECs were always significantly lower (p<0.01) in comparison to nMS patients, uMS patients, and HD. The levels of TRECs of uMS patients and HD were also significantly different (p<0.01). KRECs of the pmlMS patient were lower (p<0.01) than those of nMS and HD at T0 and T4, lower than uMS at T0, and higher than those of uMS and HD at T1, T2 and T3. Horizontal lines indicate means and error bars indicate the 99% confidence intervals of the means, calculated after log transformation of the data.

The number of TRECs and KRECs in pmlMS was compared to that in nMS, uMS, and HD. Before therapy beginning (T0), both new T and B cells were significantly lower in the pmlMS patient than in nMS and uMS patients or in HD (Figure 1). TRECs in the pmlMS patient slightly increased starting from 6 months of natalizumab therapy but then decreased to the pre-treatment level at the moment of PML diagnosis. In the other nMS patients, the pre-therapy levels of TRECs were similar to those in the uMS patients and HD, but the levels increased at 6 months of therapy and remained constantly higher than in the uMS patients for the treatment period. KRECs in the pmlMS patient peaked after 6 months of therapy and reached the values observed in the other nMS patients (in whom KRECs were significantly higher than in uMS patients and HD for the period of treatment), but the number of KRECs decreased to significantly lower values when PML developed.

### Flow cytometric analysis of T- and B-cell subpopulations

**Figure 2:**
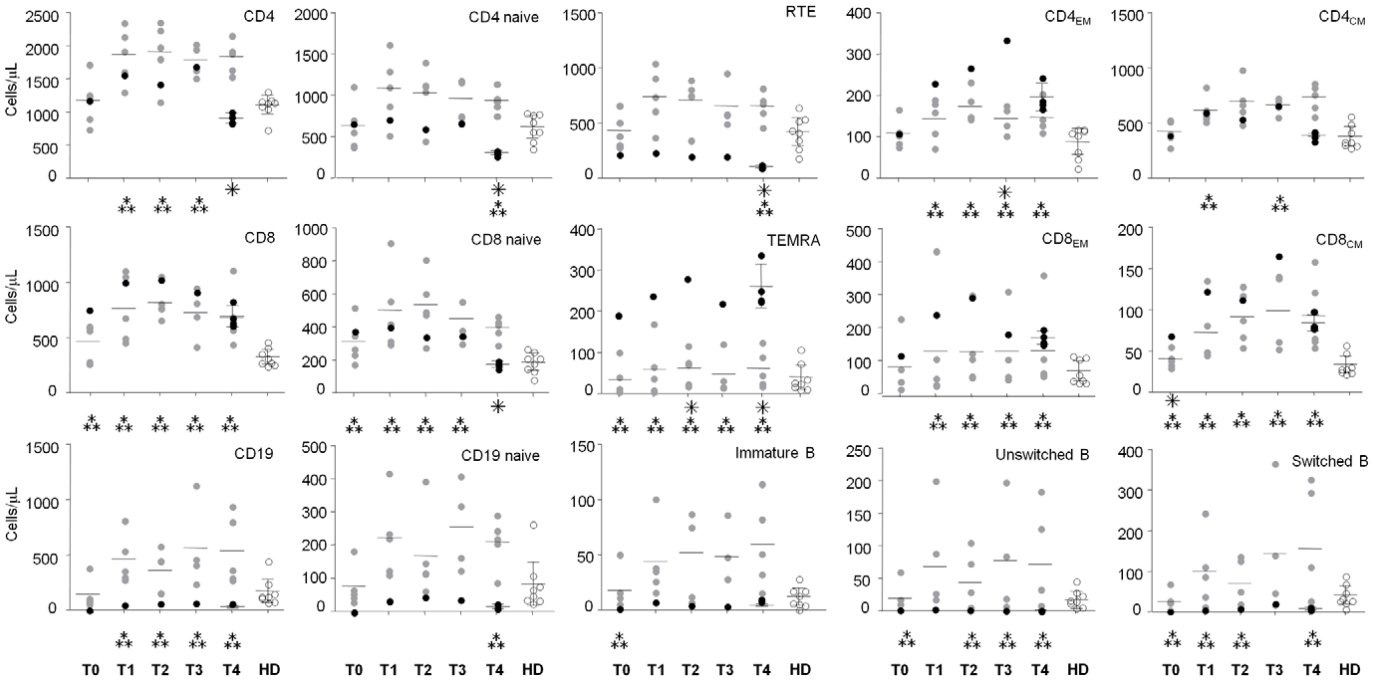
Absolute numbers of the studied cell subtypes. Absolute numbers of the indicated cell subtypes were analyzed as reported in Figure S1 and at the time points described in Table 1, of the patient who developed PML (pmlMS; black dots), natalizumab-treated patients (nMS; gray dots), and healthy donors (HD; white dots). Four samples were analyzed at the time point T4 in the pmlMS patient. ??? significant differences between cell subsets of the pmlMS patient in comparison to those of nMS patients; ??? significant differences between cell subsets of the pmlMS patient in comparison to those of HD. Horizontal lines indicate means and error bars indicate the 95% confidence intervals of the means.

By applying flow cytometry (the gating strategy used to identify the T- and B-lymphocyte populations is displayed in the Figure S1), we found an abnormal percentage and number of several T- and B-cell subsets in the pmlMS patient in comparison to nMS patients and HD. Some anomalies were observed before therapy initiation and others during therapy or after PML diagnosis. Indeed, whereas in nMS patients the percentage of all T- and B-cell subsets analyzed at all time points was not different from that in HD, a lower percentage of recent T emigrants (RTE) and naive B cells as well as a higher percentage of CD8^+^CD45RA^+^CCR7^−^ effector memory T (TEMRA) lymphocytes, and of immature and unswitched memory B cells were found in the pmlMS patient before therapy (Table S1). The totality of B-lymphocyte percentage increased in this patient at 6 months of therapy (T1), with naive B cells increasing from 0 to 71.9%. In contrast, the significantly lower percentage of RTE and the high frequency of TEMRA observed in the pre-treatment sample remained for the entire period of treatment and were particularly evident when PML occurred. The defects involving these two T-cell subsets were evident also when the results were expressed as cell/µL and, despite the increase of B-cell percentage, the number of all B-cell subsets remained very low for the entire study period (Figure 2). It is worth noting that there was a higher number of CD8^+^ cells observed in the pmlMS patient during the treatment period with respect to HD and a high number of CD4^+^ cells that decreased sharply at the time of PML diagnosis, leading to a reduction in the CD4/CD8 ratio that was not present in the other nMS patients.

### Analysis of cell precursors using CD34, DNTT and Vpreβ1 quantification

**Figure 3:**
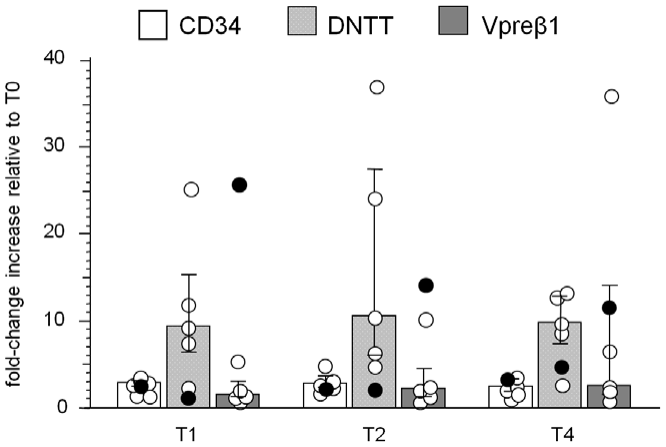
Variations of CD34, DNTT and Vpreβ1 transcript expressions. Fold-change increase of transcripts for CD34, terminal deoxynucleotidyltransferase (DNTT), expressed by lymphoid precursors, and V pre-B lymphocyte gene 1 (Vpreβ1), a part of the pre-B cell receptor in the patient who developed PML (pmlMS; black circles) and natalizumab-treated patients (nMS; white circles) were represented at the indicated time points. Bars indicate medians and error bars indicate the 25^th^ and 75^th^ percentile. Data were calculated with the ΔΔCt method using the value obtained with pre-therapy samples (T0) as the calibrator for each patient.

RNA for CD34 (a marker for early precursors that gives rise to lymphoid and myeloid cells), DNTT (a marker for lymphocyte precursors), and Vpreβ1 (an element of the pre-B-cell receptor present on immature B cells) was quantified in the samples taken at T0, T1, T2, and T4. Natalizumab induced an increase in the transcripts for all three targets in pmlMS and in nMS patients. However, while in nMS patients the increase of transcripts for DNTT (with a median of 9.5-, 10.7-, and 9.9-fold increase respectively at T1, T2, and T4 in respect to T0) was higher than that for Vpreβ1 (1.7-, 2.3- and 2.8-fold increase at T1, T2, and T4, respectively) and for CD34 (3.0- ,3.0-, and 2.6-fold increase at T1, T2, and T4, respectively), pmlMS patient showed a peak in Vpreβ1 transcript levels after 6 months of therapy, when the maximum increase in KRECs was also found (Figure 3). The increase during therapy was also evident when the level of expression of CD34, DNTT, and Vpreβ1 was calculated as a normalization ratio and compared to uMS and HD (Figure S2).

### T-cell repertoire heterogeneity analysis and Ig production

**Figure 4:**
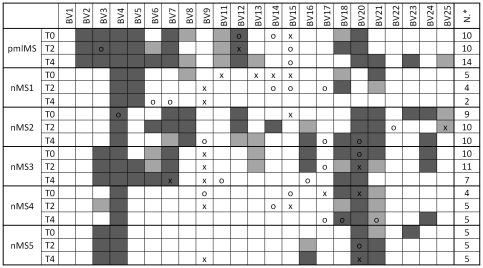
T-cell repertoire heterogeneity. Percent usage of TCRBV and perturbations of TCR CDR3 length distributions at the indicated time points in the patient who developed PML (pmlMS) and patients treated with natalizumab (nMS1 to nMS5) were determined by spectratyping analysis. The TCRBV chains were considered overused when their percent usage was higher than mean+2SD (“x") or mean+3SD (“o") of the TCRBV chain usage found in 5 healthy donors (HD). Perturbations were obtained by calculating the generalized Hamming distance according to Gorochov et al. [26] and were considered significant when their values were beyond the mean+2SD (light gray squares) or the mean+3SD (dark gray squares) of the CDR3 distribution found in HD. N*: number of perturbed TCRBV chains at each time point.

**Figure 5:**
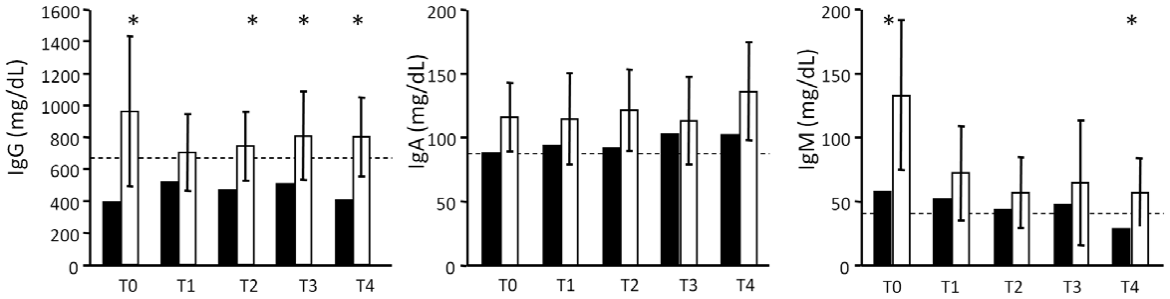
IgG, IgA, and IgM concentration. IgG, IgA, and IgM concentration was analyzed at the indicated time points in the sera of the patient who developed PML (pmlMS; black bars) and patients treated with natalizumab (nMS; white bars). Bar height represents the mean value and error bars represent the 95% confidence interval of the mean. * Measures of the pmlMS patient that were outside the 95% confidence interval of the means found in nMS. Dashed lines represent the lowest limit of the laboratory reference interval for healthy subjects.

Because it has been demonstrated that defects of new T-lymphocyte production result in a low T-cell heterogeneity [25], [27], [28], we analyzed the TCRBV chain repertoire of natalizumab-treated patients by spectratyping analysis, a technique that allowed us to assess the composition of a T-cell population and its overall diversity based on the TCRBV CDR3 length distribution. As previously reported [29], the TCRBV9 chain is one of the preferentially expressed TCRBV segments, at least at one time point of the follow up (Figure 4). All nMS patients, including the pmlMS patient, showed a more or less perturbed TCRBV repertoire (from 5 to 10 TCRBV CDR3 were skewed in comparison to CDR3 distribution observed in HD) before therapy beginning, but natalizumab induced a further strong restriction of the TCR repertoire only in the pmlMS patient. Similarly, to investigate whether anomalies of the B-cell compartment affected humoral immunity, we measured the concentrations of IgA, IgG, and IgM. In all samples of the pmlMS patient, IgG were under the lowest levels observed in HD. They were also significantly lower than those in nMS patients, with the only exception being the sample obtained at T1 (Figure 5). IgM were under the cut-off level only at the moment of PML and were significantly lower than in the other nMS patients in the pre-therapy sample. Moreover, oligoclonal bands were found in the CSF both before natalizumab treatment and after PML diagnosis. Quantification of free Ig light chains was found to be normal and without alteration of the kappa/lambda ratio in serum and CSF.

## Discussion

Currently, there are no specific ways to predict which natalizumab-treated patients are at risk for PML, except for the length of the therapy, the presence of anti-JCV antibodies, and a previous therapy with immunosuppressors [30]. Tests for JCV detection in blood or urine do not provide predictive information [31], and there are no defined immune abnormalities that can be used predictively. Furthermore, concerning the pathogenesis of PML, there are only a few certainties; the relative contributions of risk factors, including the role of a biological agent used to treat a disorder in PML development, are still unknown and, similarly, it is still incompletely understood how the virus infects the CNS. Indeed, after primary infection, JCV persists in the kidneys of healthy individuals [32]; there might be reactivations of the latent forms present in the kidneys [33], tonsils [34], and lymphoid progenitors [9], but the virus probably infects the CNS only in the case of alteration of the immune response, as in the case of HIV infection and/or treatment with immune system-modulating compounds. Alternatively, the virus primarily infects the CNS [35] and persists there for many years. These two possibilities are not mutually exclusive. Once JCV is in the CNS, it may be controlled by T cells that migrate into the CNS; in the case of an altered immune response, this control might be reduced and full blown viral infection can emerge. Thus far, the results concerning the immune response during natalizumab therapy are conflicting [21], [22], and a case of PML has also been described in an apparently immunocompetent patient [36]. Here, we demonstrate that in the patient who developed PML, some immune cell alterations were already present before therapy initiation, some were induced by the therapy and others developed at the time of PML diagnosis. In particular, in this patient, TRECs and KRECs were detected at very low levels before natalizumab treatment. This patient's age was similar to that of three other natalizumab-treated patients who, however, showed much higher number of TRECs (Figure S3); this suggests that the TREC dependency on age [37], which is even more prominent in MS [38], does not explain the low TRECs observed in pmlMS patient. Our results should not be biased by any residual effects of previous immunosuppressants because, while it has been reported that mitoxantrone apparently persists in the body for as long as 272 days [39], the patient that developed PML stopped receiving this drug 4.5 years before starting natalizumab therapy. Furthermore, the deficit of TRECs and KRECs was not observed either in nMS3 and nMS4 patients, who had stopped mitoxantrone respectively 6 and 4 years before natalizumab initiation, or in other MS patients that had received mitoxantrone in the past (data not shown). Finally, we have not found a relationship between the mitoxantrone doses and TREC or KREC levels.

The anomalies of TRECs and KRECs were respectively confirmed by the low percentage and number of RTE, which represent the T-cell subpopulation recently released from the thymus, and by the low or absent naive and mature B cells found in the peripheral blood. Defects of T-cell production persisted in the pmlMS patient for all the treatment period but did not result in a decrease in the total CD4 and CD8 cell number, probably because of the peripheral T-cell expansion. This expansion could be sustained by CD4 T_EM_ cells, which peaked at 15 months after natalizumab treatment beginning; by CD8 lymphocytes, which were particularly high at 12 months of therapy; and by TEMRA lymphocytes, which were very high for the entire period of observation. In particular, TEMRA, which were shown to be generated upon homeostatic proliferation in the absence of antigen [40] and described to be the mediators of inflammation and cytotoxicity, were also found to be up-modulated during the treatment of MS patients with fingolimod, a drug that reduces the recirculation of proinflammatory T cells to the periphery [41]. The presence of a peripheral expansion of T cells was confirmed by the skewed T-cell repertoire observed in the pmlMS patient and by the further reduction in TCRBV diversity at the moment of PML diagnosis.

The release of KREC^+^ cells from the bone marrow increased dramatically at 6 months of therapy, when KRECs reached the values observed in the other nMS patients, but after PML, it suddenly decreased to levels lower than those of HD. KREC increase is sustained by the induction of transcripts present in lymphocyte precursors, especially of Vpreβ1, which is selectively expressed at the early stages of B-cell development, namely, in pro-B and early pre-B cells [42]. Indeed, among the marker transcripts expressed during lymphocyte development, Vpreβ1 was the most increased in pmlMS patient in comparison to pre-therapy level, while, as already reported by Krumbholz et al [19], DNTT transcripts were predominantly increased in nMS patients that did not develop the PML. DNTT gene encodes a DNA polymerase that catalyzes the addition of deoxynucleotides to the 3′-hydroxyl terminus of the rearranged B-cell receptor and TCR gene segments. In vivo, the encoded protein is expressed in a restricted population of pre-B and pre-T lymphocytes during early differentiation, where it generates antigen receptor diversity by synthesizing non-germ line elements at the junctions of rearranged Ig heavy chain and TCR gene segments.

The use of frozen cells is a limitation of the study because freezing might affect the flow cytometry analysis. Accordingly, while we have adapted and validated the assay for the use of cryopreserved PBMC and we have monitored the quality of the frozen cells to ensure reliable results in phenotypic assay, we have found that CD10 cell surface expression on CD19^+^ lymphocytes is decreased and more variable when thawed PBMC are analyzed (personal observation). Therefore we could not quantify the number of CD19^+^CD10^+^ pre-B cells, but it is already known that this B-cell subpopulation is highly increased in natalizumab-treated patients [19].

The increased KREC production resulted in a higher percentage of naive B-cells, whose number, however, remained very low for the entire period of the study. Accordingly, IgG were lower than in nMS patients and HD, with the only exception of the moment in which KRECs peaked. However, the lack of a significant difference in IgG levels between pmlMS and nMS patients at T1 appeared to be more related to the decreased IgG observed in nMS patients than to a hypothetical rise following the sharp increase in KREC production. Indeed, the fact that natalizumab treatment impairs the homing to the tissues [43] may interfere with the maturation of B cells and the subsequent production of Ig. Even if the ability of JCV to replicate in B lymphocytes is controversial [44], [45], the virus has been found in B cells from lymphoid tissues of PML patients [46], and seems capable to reside in cells of this lineage presumably long enough to employ them as a potential vehicle to cross the blood-brain barrier and transmit the infection to the oligodendrocytes in the brain [44]. The frequent association of PML with B-cell malignancies, or with disorders in which a widespread B-cell activation is routinely observed, may support the hypothesis that B-cell activation can lead to JCV activation in B cells productively infected by JCV. In fact, it has been demonstrated that natalizumab leads to an upregulation of cell nuclear transcription factors involved in B-cell activation and differentiation and, perhaps, in JCV expression [47], [48]. Thus, it could also potentially lead to JCV expression, replication and even genetic mutation to a neurotropic strain. Indeed, although it has been well recognized that re-arrangements in the noncoding control region are important for enabling JCV to replicate effectively in glial cells, point mutations such those of the VP1 protein have been suggested as contributors of PML development [49]. While the specific site of the JC viral genome alteration remains uncertain, the intracellular presence of the virus in cells of the B-cell lineage, which are uniquely engineered to modify the genome, strongly implicates their importance.

In conclusion, our data indicate that the profound impairment of cellular and humoral immunity in the pmlMS patient both before and during the therapy may have predisposed our patient to PML development. Furthermore, our data suggest that the assay for TRECs/KRECs may help identify MS patients at risk of developing PML. The validation of these risk candidates needs further studies in larger cohorts of prospectively followed patients undergoing long-term natalizumab therapy.

## Supporting Information

Figure S1 **The gating strategy used to identify the T- and B-lymphocyte populations.** PBMCs were stained with the indicated fluorochrome-conjugated MoAb and B- and T-cell subsets were identified. **A.** CD3^+^ cells were first gated on lymphocytes and then analyzed for the expression of CD4 and CD8 markers, which in turn were gated and analyzed for the expression of CD45RA and CCR7 in order to identify CD45RA^+^CCR7^+^ naive lymphocytes, CD45RA^−^CCR7^+^ central memory (T_CM_), and CD45RA^−^CCR7^−^ effector memory (T_EM_) T cells, as well as CD8^+^CD45RA^+^CCR7^−^ effector memory T lymphocytes (TEMRA). Furthermore, the expression of CD31 on naive CD4^+^ cells was used to recognize recent T emigrants (RTE), which are T lymphocytes that have been recently released from the thymus. **B.** CD19^+^ cells were first gated on lymphocytes and then analyzed for the expression of CD21 marker that identifies CD19^+^CD21^low/−^ immature B cells and CD19^+^CD21^+^ mature B cells, which in turn were gated and analyzed for IgD and CD27 molecule expression in order to recognize IgD^+^CD27^−^ naive B cells, IgD^+^CD27^+^ unswitched memory B cells, and IgD^−^CD27^+^ switched memory B cells. In each panel, the percentages of cell subsets of a representative healthy donor are shown.
(TIF)

Figure S2 **RNA expression of CD34, terminal deoxynucleotidyltransferase (DNTT), and V pre-B lymphocyte gene 1 (Vpreβ1) transcripts.** RNA expression was quantified at the indicated time points by real-time PCR in the patient who developed PML (pmlMS; black circles) and in the 5 patients treated with natalizumab (nMS1 to nMS5; white symbols) and reported as normalization ratio (NR), relative to the same subject, who is used as calibrator. Means and error bars indicating the 95% confidence intervals of data obtained in untreated MS patients (uMS) and healthy donors (HD) are shown on the right.
(TIF)

Figure S3 **Correlation between TRECs and age.** TRECs/mL were plotted against the age in the untreated MS patients (uMs), healthy donors (HD), and in the follow-up time-points of the patient who developed PML(pmlMS) and of those treated with natalizumab (nMS). Grey line (HD) and black line (uMS) were obtained by linear regression showing a similar age-related TREC decrease in the two groups, with uMS patients, however, whose TRECs were significantly lower (intercept comparison: p = 0.01).
(PDF)

Table S1 **Percentage of the indicated lymphocyte subtypes.**
(DOC)
