# Supplementary figures and images for: Pre-Existing T- and B-Cell Defects in One Progressive Multifocal Leukoencephalopathy Patient
Source: PLoS One. 2012 Apr 4;7(4):e34493. doi: 10.1371/journal.pone.0034493 (PMC3319584; doi:10.1371/journal.pone.0034493)

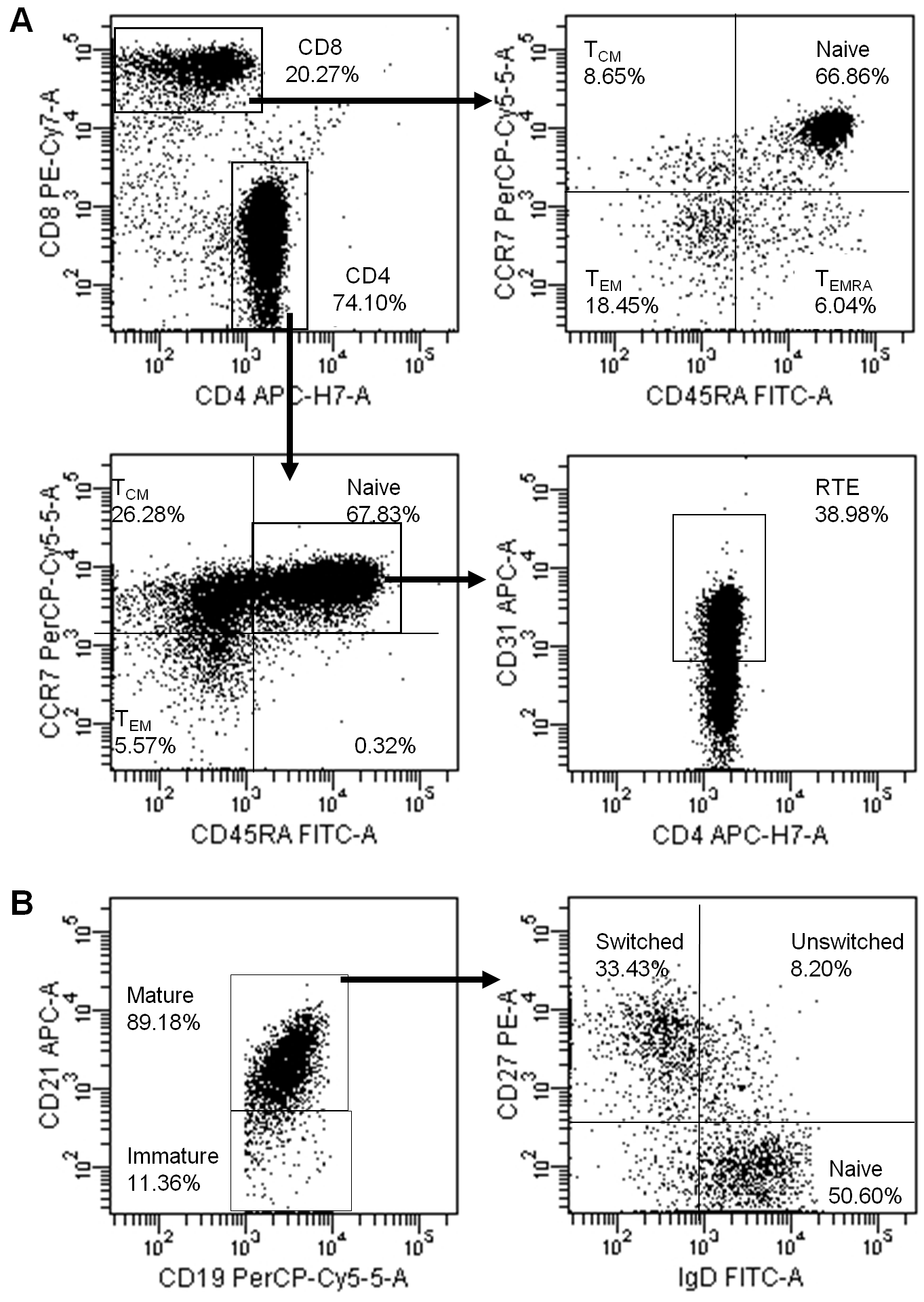

Supplement: Figure S1 — The gating strategy used to identify the T- and B-lymphocyte populations. PBMCs were stained with the indicated fluorochrome-conjugated MoAb and B- and T-cell subsets were identified. A. CD3+ cells were first gated on lymphocytes and then analyzed for the expression of CD4 and CD8 markers, which in turn were gated and analyzed for the expression of CD45RA and CCR7 in order to identify CD45RA+CCR7+ naive lymphocytes, CD45RA−CCR7+ central memory (TCM), and CD45RA−CCR7− effector memory (TEM) T cells, as well as CD8+CD45RA+CCR7− effector memory T lymphocytes (TEMRA). Furthermore, the expression of CD31 on naive CD4+ cells was used to recognize recent T emigrants (RTE), which are T lymphocytes that have been recently released from the thymus. B. CD19+ cells were first gated on lymphocytes and then analyzed for the expression of CD21 marker that identifies CD19+CD21low/− immature B cells and CD19+CD21+ mature B cells, which in turn were gated and analyzed for IgD and CD27 molecule expression in order to recognize IgD+CD27− naive B cells, IgD+CD27+ unswitched memory B cells, and IgD−CD27+ switched memory B cells. In each panel, the percentages of cell subsets of a representative healthy donor are shown. (TIF) [file pone.0034493.s001.tif]

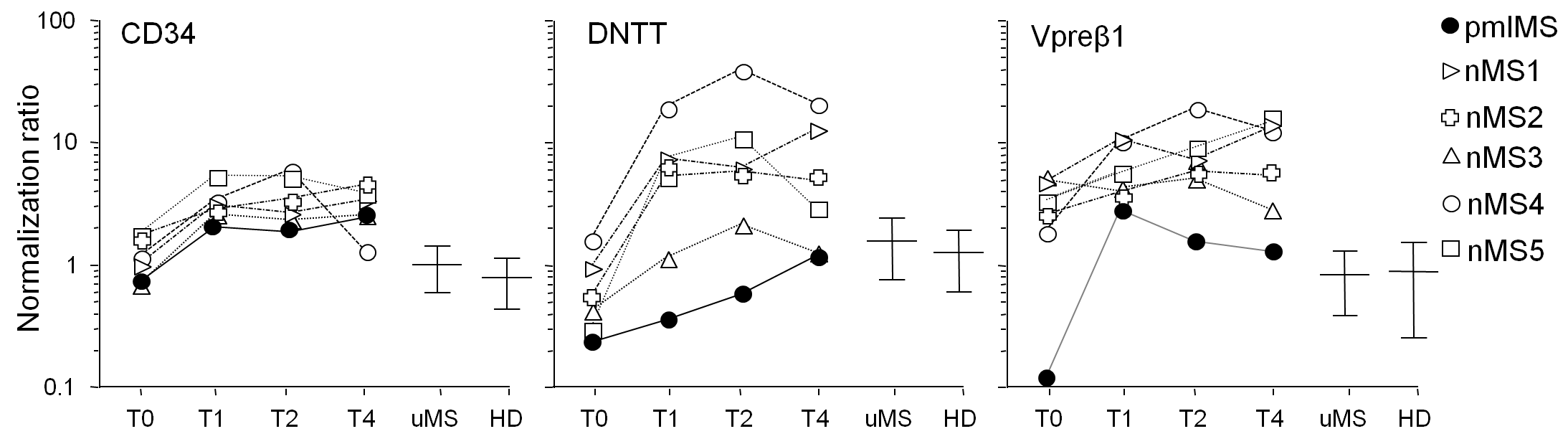

Supplement: Figure S2 — RNA expression of CD34, terminal deoxynucleotidyltransferase (DNTT), and V pre-B lymphocyte gene 1 (Vpreβ1) transcripts. RNA expression was quantified at the indicated time points by real-time PCR in the patient who developed PML (pmlMS; black circles) and in the 5 patients treated with natalizumab (nMS1 to nMS5; white symbols) and reported as normalization ratio (NR), relative to the same subject, who is used as calibrator. Means and error bars indicating the 95% confidence intervals of data obtained in untreated MS patients (uMS) and healthy donors (HD) are shown on the right. (TIF) [file pone.0034493.s002.tif]

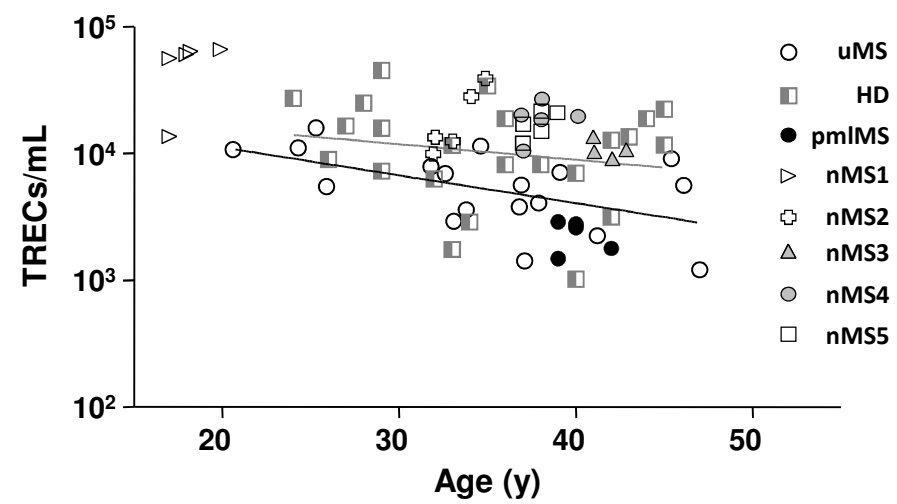

Supplement: Figure S3 — Correlation between TRECs and age. TRECs/mL were plotted against the age in the untreated MS patients (uMs), healthy donors (HD), and in the follow-up time-points of the patient who developed PML(pmlMS) and of those treated with natalizumab (nMS). Grey line (HD) and black line (uMS) were obtained by linear regression showing a similar age-related TREC decrease in the two groups, with uMS patients, however, whose TRECs were significantly lower (intercept comparison: p = 0.01). (PDF) [file pone.0034493.s003.pdf]
