# Supplementary material for: Pre-Existing T- and B-Cell Defects in One Progressive Multifocal Leukoencephalopathy Patient
Source: PLoS One. 2012 Apr 4;7(4):e34493. doi: 10.1371/journal.pone.0034493 (PMC3319584; doi:10.1371/journal.pone.0034493)
Supplement: Table S1 — Percentage of the indicated lymphocyte subtypes. (DOC) [file pone.0034493.s004.doc]

| **Table S1. Percentage of the indicated lymphocyte subtypes.** | | | | | | | | | | | |  |  | |  | | | | | |  | | | |  | |  | | | |  | |  |  |
| --- | --- | --- | --- | --- | --- | --- | --- | --- | --- | --- | --- | --- | --- | --- | --- | --- | --- | --- | --- | --- | --- | --- | --- | --- | --- | --- | --- | --- | --- | --- | --- | --- | --- | --- |
|  |  |  |  |  | **T0** | |  | **T1** | |  | **T2** | |  | | **T3** | | | | | | | | | |  | | **T4** | | | | | |  |  |
|  |  |  | **Cell subsets** |  | **pmlMS** | **nMS** |  | **pmlMS** | **nMS** |  | **pmlMS** | **nMS** |  | | **pmlMS** | | | | | | **nMS** | | | |  | | **pmlMS** | | | | **nMS** | |  | **HD** |
|  |  |  |  |  |  |  |  |  |  |  |  |  |  | |  | | | | | |  | | | |  | |  | | | |  | |  |  |
| **T lymphocytes** |  |  | CD4 (% of CD3) | mean | 58.5* | 64.9 |  | 58.7* | 66.5 |  | 56.0* | 65.0 |  | | 59.7* | | | | | | 66.6 | | | |  | | 53.8* | | | | 67.9 | |  | 70.3 |
|  |  |  |  | sd |  | 9.4 |  |  | 10.7 |  |  | 7.4 |  | |  | | | | | | 8.8 | | | |  | |  | | | | 8.1 | |  | 5.8 |
|  |  |  | CD4 naive (% of CD4) | mean | 56.2 | 52.4 |  | 45.8 | 56.4 |  | 42.1 | 52.8 |  | | 39.5 | | | | | | 53.4 | | | |  | | 33.9* | | | | 51.3 | |  | 55.7 |
|  |  |  |  | sd |  | 8.7 |  |  | 12.0 |  |  | 9.3 |  | |  | | | | | | 6.8 | | | |  | |  | | | | 4.9 | |  | 10.6 |
|  |  |  | RTE (% of CD4) | mean | 18.3*° | 36.6 |  | 15.0* | 38.9 |  | 14.2*° | 36.9 |  | | 12* | | | | | | 36.6 | | | |  | | 11.6*° | | | | 36.0 | |  | 37.5 |
|  |  |  |  | sd |  | 4.2 |  |  | 9.5 |  |  | 6.3 |  | |  | | | | | | 9.5 | | | |  | |  | | | | 7.2 | |  | 11.3 |
|  |  |  | CD4 T_EM_ (% of CD4) | mean | 9.2 | 9.9 |  | 14.8* | 8.2 |  | 18.8* | 9.7 |  | | 19.8* | | | | | | 8.3 | | | |  | | 21.6*° | | | | 8.1 | |  | 7.9 |
|  |  |  |  | sd |  | 3.3 |  |  | 4.1 |  |  | 3.9 |  | |  | | | | | | 2.9 | | | |  | |  | | | | 1.7 | |  | 3.0 |
|  |  |  | CD4 T_CM_ (% of CD4) | mean | 33.2 | 36.9 |  | 38.2 | 33.6 |  | 37.8 | 36.8 |  | | 39.0 | | | | | | 37.3 | | | |  | | 43.0 | | | | 40.0 | |  | 34.7 |
|  |  |  |  |  |  | 5.3 |  |  | 6.0 |  |  | 5.4 |  | |  | | | | | | 4.1 | | | |  | |  | | | | 3.4 | |  | 9.1 |
|  |  |  |  |  |  |  |  |  |  |  |  |  |  |  |  |  |  |  |  |  |  |  |  |  |  |  |  |  |  |  |  |  |  |  |
|  |  |  | CD8 (% of CD3) | mean | 37.2* | 25.7 |  | 37.7* | 26.8 |  | 40.4* | 28.5 |  | | 32.3* | | | | | | 26.5 | | | |  | | 40.9* | | | | 24.7 | |  | 20.8 |
|  |  |  |  | sd |  | 7.3 |  |  | 9.0 |  |  | 5.7 |  | |  | | | | | | 6.7 | | | |  | |  | | | | 6.8 | |  | 3.8 |
|  |  |  | CD8 naive (% of CD8) | mean | 49.8 | 68.3 |  | 40.0 | 68.7 |  | 33.4 | 65.2 |  | | 37.9 | | | | | | 64.7 | | | |  | | 25.6*° | | | | 61.5 | |  | 58.1 |
|  |  |  |  | sd |  | 16.5 |  |  | 23.1 |  |  | 18.2 |  | |  | | | | | | 17.3 | | | |  | |  | | | | 12.4 | |  | 17.9 |
|  |  |  | CD8 TEMRA (% of CD8) | mean | 25.5*° | 6.4 |  | 23.8* | 7.5 |  | 27.2*° | 7.8 |  | | 24.1* | | | | | | 5.9 | | | |  | | 37.6*° | | | | 8.6 | |  | 11.3 |
|  |  |  |  | sd |  | 6.8 |  |  | 7.3 |  |  | 5.2 |  | |  | | | | | | 4.7 | | | |  | |  | | | | 4.8 | |  | 7.4 |
|  |  |  | CD8 T_EM_ (% of CD8) | mean | 15.6 | 15.7 |  | 23.9 | 14.1 |  | 28.5 | 15.6 |  | | 19.9 | | | | | | 15.9 | | | |  | | 24.6 | | | | 16.4 | |  | 20.4 |
|  |  |  |  | sd |  | 13.3 |  |  | 15.3 |  |  | 12.4 |  | |  | | | | | | 11.3 | | | |  | |  | | | | 9.2 | |  | 9.2 |
|  |  |  | CD8 T_CM_ (% of CD8) | mean | 9.2 | 9.7 |  | 12.3 | 9.8 |  | 11.0 | 11.5 |  | | 18.1* | | | | | | 13.5 | | | |  | | 12.3 | | | | 13.4 | |  | 10.3 |
|  |  |  |  |  |  | 4.0 |  |  | 3.3 |  |  | 4.2 |  | |  | | | | | | 4.0 | | | |  | |  | | | | 3.2 | |  | 4.2 |
| **B lymphocytes** |  |  |  |  |  |  |  |  |  |  |  |  |  | |  | | | | | |  | | | |  | |  | | | |  | |  |  |
|  |  |  | CD19 (% of lymphocytes) | mean | 0.3* | 6.4 |  | 1.6* | 10.2 |  | 1.8 | 8.4 |  | | 2.0 | | | | | | 12.3 | | | |  | | 1.2* | | | | 12.2 | |  | 7.7 |
|  |  |  |  | sd |  | 6.6 |  |  | 4.2 |  |  | 4.6 |  | |  | | | | | | 5.4 | | | |  | |  | | | | 6.3 | |  | 4.0 |
|  |  |  | IMMATURE (% of CD19 ) | mean | 31.8*° | 11.6 |  | 14.4 | 9.2 |  | 7.1 | 12.3 |  | | 6.0 | | | | | | 9.2 | | | |  | | 14.8 | | | | 11.0 | |  | 8.9 |
|  |  |  |  | sd |  | 5.8 |  |  | 3.7 |  |  | 5.5 |  | |  | | | | | | 2.4 | | | |  | |  | | | | 4.3 | |  | 4.3 |
|  |  |  | NAIVE (% of MATURE) | mean | 0*° | 62.5 |  | 71.9 | 55.8 |  | 76.7* | 54.5 |  | | 54.1 | | | | | | 54.8 | | | |  | | 55.4 | | | | 52.4 | |  | 50.1 |
|  |  |  |  | sd |  | 18.6 |  |  | 24.8 |  |  | 24.1 |  | |  | | | | | | 20.4 | | | |  | |  | | | | 28.1 | |  | 15.6 |
|  |  |  | MEMORY UNSWITCHED (% of MATURE) | mean | 45.5*° | 10.9 |  | 6.9 | 14.1 |  | 2.6 | 14.4 |  | | 1.8* | | | | | | 12.6 | | | |  | | 2.1* | | | | 11.6 | |  | 13.6 |
|  |  |  |  | sd |  | 8.5 |  |  | 12.6 |  |  | 11.5 |  | |  | | | | | | 8.6 | | | |  | |  | | | | 9.1 | |  | 7.4 |
|  |  |  | MEMORY SWITCHED (% of MATURE) | mean | 54.5* | 18.8 |  | 16.1 | 23.4 |  | 17.5 | 23.9 |  | | 34.0° | | | | | | 24.3 | | | |  | | 33.7 | | | | 28.4 | |  | 27.7 |
|  |  |  |  |  |  | 13.4 |  |  | 15.5 |  |  | 15.7 |  | |  | | | | | | 14.0 | | | |  | |  | | | | 18.9 | |  | 9.8 |
|  |  |  |  |  |  |  |  |  |  |  |  |  |  | |  | | | | | |  | | | |  | |  | | | |  | |  |  |
| The analysis was performed at the indicated time points (T0: pretreatment samples; for T1, T2, T3, and T4: see Table 1) | | | | | | | | | | | | | | | | | | | | | | | | | | | | |  | | |  | | |
| * Values of patient with PML (pmlMS) which lie outside 95% confidence interval of HD | | | | | | | | | | | | | | | | | | | |  | |  |  |  | |  | |  | |  | |  | | |
| ° Values of patient with PML (pmlMS) which lie outside 95% confidence interval of natalizumab-treated MS patients (nMS) | | | | | | | | | | | | | | | | | | | | | | | | | | | | |  | | |  | | |
|  | | | | | | | | | | | | | |  | |  |  |  |  |  | |  |  |  | |  | |  | |  | |  | | |
